# Association between Changes in Total Antioxidant Levels and Clinical Symptom Improvement in Patients with Antipsychotic-Naïve First-Episode Schizophrenia after 3 Months of Risperidone Monotherapy

**DOI:** 10.3390/antiox11040646

**Published:** 2022-03-28

**Authors:** Keqiang Wang, Meihong Xiu, Xiuru Su, Fengchun Wu, Xiangyang Zhang

**Affiliations:** 1 Hebei Province Veterans Hospital, Baoding 071000, China; kqwang79@163.com (K.W.); xiuxiususu321097@163.com (X.S.); 2 Beijing HuiLongGuan Hospital, HuiLongGuan Clinical Medical School, Peking University, Beijing 100096, China; xiumeihong97@163.com; 3 Department of Psychiatry, The Affiliated Brain Hospital of Guangzhou Medical University, Guangzhou 510370, China; 4 Department of Biomedical Engineering, Guangzhou Medical University, Guangzhou 510370, China; 5 Guangdong Engineering Technology Research Center for Translational Medicine of Mental Disorders, Guangzhou 510370, China; 6 CAS Key Laboratory of Mental Health, Institute of Psychology, Chinese Academy of Sciences, Beijing 100083, China

**Keywords:** schizophrenia, antioxidant protection, therapeutic response, antipsychotics

## Abstract

Schizophrenia (SCZ) is associated with aberrant redox regulation in the early stages of brain development. There is growing evidence that the antioxidant defense system is closely associated with the therapeutic response to antipsychotics in SCZ patients. The aim of this study was to examine the effect of risperidone monotherapy on total antioxidant status (TAS) and the relationship between symptom improvement and changes in TAS in patients with antipsychotic-naïve first-episode (ANFE) SCZ. Clinical symptoms were evaluated using the Positive and Negative Syndrome Scale (PANSS). Two hundred and forty-six ANFE patients were treated with risperidone for 3 months. PANSS and TAS levels were assessed at baseline and at a 3-month follow-up. Relative to healthy controls, ANFE patients had higher TAS levels, which increased even further during the treatment. Moreover, baseline TAS levels were a predictor of symptom reduction after risperidone treatment. In addition, there was a significant association between increased TAS levels and the decreased cognitive factor. Our findings suggest that antioxidant protection is possibly associated with clinical improvement in ANFE patients after risperidone treatment.

## 1. Introduction

Schizophrenia (SCZ) is a chronic and debilitating disorder with a prevalence of approximately 1.3% [1]. However, the underlying pathophysiology of SCZ remains unclear. Although antipsychotic treatment can improve clinical symptoms in patients with psychosis, limited treatment based on biological mechanisms hinders the full benefits of antipsychotics. A growing number of studies have revealed multiple biochemical pathways related to neurodevelopmental abnormalities and adult brain function that may be involved in the pathophysiology of SCZ [2].

Recent data suggest that the underlying pathological mechanisms of SCZ may involve imbalances in the redox regulatory system and oxidative stress during a critical period of early brain development [3,4,5]. Any imbalance in this mechanism, as well as neuroinflammation, N-methyl-D-aspartate (NMDA) receptor hypofunction, and dopamine dysregulation, can lead to changes in the brain, such as to parvalbumin interneurons and white matter, leading to clinical symptoms [6]. In SCZ patients, researchers found that excess free radicals are produced, at least in part, by enhanced neurotransmitter metabolism and that the antioxidant defense system is upregulated to counteract free radicals [7]. Abnormal oxidative stress parameters and antioxidant defense levels were reported in peripheral samples, as well as in the brain of SCZ patients [8]. The antioxidant defense system includes enzymatic defense systems, such as catalase (CAT) and superoxide dismutase (SOD), and nonenzymatic systems, such as uric acid, vitamin C, glutathione, β-carotene, and albumin [9]. Although the concentrations of individual antioxidants can be measured separately, these measurements are time-consuming and expensive. Since the antioxidant capacity of antioxidant components is additive, the level of total antioxidant status (TAS) can be considered as a useful indicator of the sum of all antioxidants in the body [10].

Recent studies identified abnormal plasma TAS levels in ANFE SCZ patients [3]. Xie et al. found that decreased TAS levels were inversely associated with cognitive functions such as emotion regulation, attention/vigilance, and speed of processing [4]. Li et al. reported that reduced TAS levels were linked to a higher negative subscore of the Positive and Negative Syndrome Scale (PANSS) [5]. Notably, TAS has been associated not only with psychotic symptoms, but also with side effects in SCZ patients. For example, Wu et al. found that decreased TAS levels were associated with the development of tardive dyskinesia (TD) in chronically treated SCZ patients [6]. A recent study reported that electroconvulsive therapy (ECT) treatment significantly increased TAS levels and improved symptoms in SCZ patients [7]. Taken together, these studies collectively corroborate the critical role of TAS in the onset and progression of clinical symptoms in SCZ patients and suggest that it can be used as a potential biomarker for treatment prediction. Antipsychotic drugs can effectively alleviate some psychiatric symptoms in SCZ patients, at least in part by regulating oxidative stress responses [3]. Several studies have shown that antipsychotic drugs can modulate and regulate TAS levels, but the results are contradictory. For example, Virit et al. reported a significant negative association between TAS levels and symptom severity, without significant differences in TAS levels when chronic patients were treated with typical or atypical antipsychotics or a combination of both [5]. Al-Chalabi et al. reported that olanzapine monotherapy for 8 weeks increased TAS levels and partially eliminated the adverse effects of the antioxidant defense system in SCZ patients [8]. Zhang et al. have found that TAS levels were not associated with the type and dose of antipsychotic drugs [9,10].

Risperidone is a common atypical antipsychotic and the most widely used antipsychotic in China for the treatment of SCZ patients. Compared to typical antipsychotics, it is more effective in treating negative symptoms and cognitive impairment [10]. To date, to the best of our knowledge, no studies have investigated changes in TAS levels in ANFE SCZ patients during risperidone treatment. Based on the results from previous studies regarding the relationship between TAS levels, pathophysiology, and antipsychotic treatment in SCZ, we hypothesized that risperidone monotherapy would have an effect on TAS levels and that changes in TAS levels after 3 months of risperidone treatment would be associated with an improvement in clinical symptoms in ANFE SCZ patients. Therefore, we aimed to explore whether (1) TAS levels were different in ANFE SCZ patients compared with healthy controls; (2) there was a change in TAS levels after 3 months of risperidone treatment; (3) changes in TAS levels during treatment were associated with an improvement in clinical symptoms; and (4) baseline TAS levels were a predictor of symptom improvement in SCZ patients.

## 2. Materials and Methods

### 2.1. Participants

We recruited two hundred and forty-six ANFE SCZ patients from two psychiatric research centers in China. Patients were diagnosed at baseline on the basis of the Structured Clinical Interview I for DSM-IV (SCID-I) criteria. Inclusion criteria included: age 16–45 years; duration of illness <60 months; and cumulative use of antipsychotic drugs <2 weeks. Exclusion criteria included: major medical comorbidities; past history of autoimmune diseases, allergies, hypertension, and/or diabetes; cerebrovascular diseases; and pregnancy or breastfeeding. ANFE SCZ patients had their diagnosis confirmed at follow-up by DSM-IV criteria. A total of 118 unrelated healthy control (HC) subjects were recruited during the same period and screened by SCID interview to exclude potential psychiatric disorders, as described in our previous study [11]. Moreover, we obtained a complete set of medical history, physical examination, electroencephalogram (EEG) examination, and laboratory tests from patients and HCs. Participants were excluded if they had somatic diseases (i.e., infection, diabetes, and hypertension), neurological disorders, or were taking over-the-counter antioxidants. In addition, HCs were assessed for personal history of psychiatric disorders based on the standardized diagnostic assessment of the SCID. If an HC subject had a first-degree relative with a diagnosis of psychiatric disorder, they were excluded. All participants were Han Chinese. This study was approved by the Ethical Committee of Beijing Huilongguan Hospital (Ethics No. 2013-10, 15 October 2013). All participants provided written informed consent prior to enrollment.

### 2.2. Assessment of Psychiatric Symptoms and Clinical Outcomes

SCZ patients received a flexible dose of risperidone monotherapy (4~6 mg/day) for 12 weeks, following a 1-week stabilization phase after admission. After a training course on how to use the PANSS, six psychiatrists assessed psychiatric symptoms using this scale [12]. In this study, a 5-factor model, consisting of positive, negative, disorganization, excitement, and depressive factors, was used to capture the validity of the clinical symptom dimension. This model allows for a broader and multidimensional representation of patients’ clinical symptoms [12]. PANSS was evaluated at baseline and at a 3-month follow-up.

### 2.3. Determination of TAS in the Patients and Controls

Following an overnight fast, blood samples from patients and HCs were obtained in EDTA-coated tubes, which were then aliquoted and stored at −80 °C. Plasma TAS was measured by two technicians, who were blinded to the diagnosis of the participants, using a commercially available kit (Nanjing Jiancheng Bioengineering Inc., Nanjing, China) [13]. Briefly, TAS levels were evaluated as a reduction product from Fe^3+^ to Fe^2+^, which was chelated by 2,4,6-tri-pyridyl-s-triazine (TPTZ) to form an Fe^2+^-TPTZ complex that was absorbed at 593 nm and recorded using a Multiskan microplate reader (FlowLabs, McLean, VA, USA). To minimize the decay of plasma TAS after several years of recruiting patients, we measured TAS activity every six months in this study.

### 2.4. Statistical Analysis

The Shapiro–Wilk test showed that all variables in this study were normally distributed. An *X*^2^ test and analysis of variance (ANOVA) for categorical and continuous variables were used to compare demographic variables between patients and controls. Analysis of covariance (ANCOVA) was performed to examine whether there were differences in TAS levels between controls and patients at baseline. Since sex and age are closely associated with oxidative stress markers, these variables were controlled for in the MANCOVA analysis.

The last observation carried forward (LOCF) method was performed on patients who dropped out after the second month. We included 246 patients with baseline TAS levels in our study. A paired *t*-test was used to compare baseline and follow-up data. The association between TAS levels and disease severity in patients was calculated by Pearson’s correlation coefficients. Multiple linear regression analysis was conducted to determine independent factors associated with symptom improvement. Changes in PANSS and biomarkers between follow-up and baseline were computed by subtracting the baseline values from the follow-up values. All analyses were conducted in SPSS statistical analysis software (version 22.0; SPSS Inc., Chicago, IL, USA) with two-tailed statistical tests (*p* < 0.05). Using the G*Power 3.1.9.2 program, the sample size was calculated based on the effect size derived from a previous study [11]. Bonferroni correction was used to adjust for multiple tests. To obtain Bonferroni-corrected/adjusted *p* values, we divided the original alpha values by the number of analyses performed on the variables. In this study, the new α = 0.05/5 = 0.01.

## 3. Results

### 3.1. Demographic and Clinical Data of ANFE Patients at Baseline

Based on the effect size of a previous study [11], the sample size should be 140. Therefore, the 246 patients in this study were sufficient to obtain a power of 0.95 at α < 0.05 (two-tailed). Table 1 shows the demographic and baseline clinical characteristics of the patients. Among them, there was a significant correlation between TAS levels and BMI (r = −0.18, *p* = 0.006) and education (r = −0.29, *p* < 0.001). In HCs, there was no significant association between TAS levels and age (r = −0.20, *p* = 0.05), education (r = 0.07, *p* = 0.50), or BMI (r = −0.10, *p* = 0.35). Relative to HCs, ANFE patients showed higher baseline TAS levels after controlling for age and sex (223.7 ± 67.7 vs. 205.7 ± 53.5, *p* < 0.05) (Table 2).

### 3.2. Comparisons of TAS before and after Risperidone Treatment

After 3 months of treatment, the PANSS total and its subscores significantly decreased (all *p*_Bonferroni_ < 0.01). In addition, with regard to the five-factor model, risperidone treatment significantly reduced all five factors of the PANSS (all *p*_Bonferroni_ < 0.01). Risperidone monotherapy for 3 months significantly increased TAS levels (t = −7.7, *p* < 0.001) (Table 3).

### 3.3. Relationship between Baseline TAS, Changes in TAS, and Improvement in Clinical Symptoms

At baseline, TAS levels were negatively correlated with the positive factor (r = −0.21, *p* = 0.001) and excitement factor (r = −0.24, *p* < 0.001) (all *p*_Bonferroni_ < 0.05). Further multiple regression analysis confirmed the association between baseline TAS levels and the positive factor (t = −2.9, *p* = 0.005) and excitement factor (t = −3.5, *p* = 0.001).

**Figure 1:**
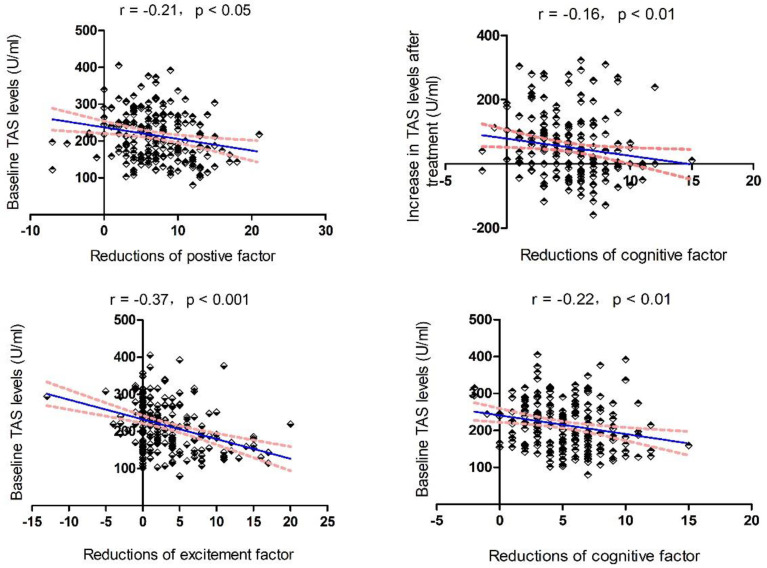
Associations between TAS levels and cognitive improvements. The red line is the trend line and the blue dashed line is the 95% confidence interval.

Moreover, baseline TAS levels significantly correlated with reductions in the positive factor (r = −0.21, *p* = 0.003), cognitive factor (r = −0.22, *p* = 0.002), and excitement factor (r = −0.37, *p* < 0.001) (Figure 1). Further multiple regression analysis revealed that baseline TAS level was a predictor of reduction in the positive factor (β = −0.186, t = −2.415, *p* = 0.02), cognitive factor (β = −0.196, t = −2.538, *p* = 0.01), and excitement factor after risperidone treatment (β = −0.345, t = −4.728, *p* < 0.001).

In addition, we found a significant association between increased TAS levels and a decreased cognitive factor (r = −0.16, *p* = 0.02), which was further confirmed by a linear regression analysis using age, education, sex, and smoking status as covariates (β = −0.143, t = −2.011, *p* = 0.046).

## 4. Discussion

This is the first and largest study to evaluate changes in TAS levels in ANFE patients after 3 months of risperidone treatment. Our results showed that (1) plasma TAS levels were higher in ANFE SCZ patients than in HCs; (2) TAS levels significantly increased after 3 months of risperidone monotherapy; and (3) baseline TAS levels were associated with a decrease in positive, cognitive, and excitement factors after risperidone monotherapy.

Our results of increased TAS levels in ANFE SCZ patients are in line with those of some previous studies [14]. Because TAS represents the general antioxidant defense capacity of all antioxidants in individuals, the elevated TAS levels in ANFE patients suggest an abnormality in the antioxidant defense system in the very early stages of SCZ. A growing number of studies have demonstrated that SCZ is associated with persistent redox dysregulation [15]. Many studies have consistently reported increased oxidative stress in SCZ patients who have never received antipsychotic treatment [13]. We hypothesize that, as a compensatory mechanism, the elevated TAS levels in this study may indicate an overstressed antioxidant status meant to protect against oxidative damage. However, several studies have reported inconsistent and conflicting results [14,15,16]. For example, Mico et al. found that a group of adolescents with early-onset and first-episode psychosis had lower TAS levels than healthy controls [17]. A possible reason for this discrepancy may lie in the age of the participants and the different stages of disease progression. Our study recruited adult ANFE SCZ patients with an average age of 26.9 years, whereas the average age of subjects in Mico et al.’s study was 15.6 years [17]. Taken together, these studies suggest that there are abnormalities in TAS levels in ANFE SCZ patients.

Our study further showed a significant increase in TAS levels after 3 months of risperidone monotherapy. Relative to HCs, TAS levels in ANFE patients were higher at baseline and increased further after 3 months of risperidone treatment, suggesting that TAS levels in SCZ patients changed dynamically from baseline to the end of treatment. Our results suggest that a well-regulated oxidant detoxification system that prevents oxidative stress may be overstressed during the early stages of psychosis and even more so during treatment. There is mounting evidence that antipsychotic drugs enhance the excessive production of free radicals through drug metabolism, which then further leads to an increase in oxidative stress markers [18,19]. As a compensatory mechanism, TAS levels increase with the increases in free radicals and pro-oxidants. Therefore, our findings of elevated TAS levels suggest that oxidative stress may occur during the initial stage of the disease and remain throughout the progression of the disease. Additionally, changes in TAS levels may not be a direct consequence of risperidone, but an indirect consequence of changes in the symptoms of SCZ patients. Future studies are needed in order to evaluate the effects of antipsychotic drugs on TAS levels and other oxidative stress markers in SCZ patients.

Our third finding was that baseline TAS levels were associated with the clinical phenotype of patients and predicted response to risperidone treatment. We showed that patients with lower baseline TAS levels were more likely to experience symptom relief than those with higher TAS levels, suggesting that peripheral levels of TAS are prognostic biomarkers of antipsychotic response. We hypothesize that the possible mechanism for this is related to the symptoms of oxidative stress damage in patients with low TAS levels. Therefore, treatment response to risperidone treatment is better. SCZ is a brain disorder. It is thought that the brain is a highly oxidized organ, consuming 20% of the body’s oxygen [20], and is susceptible to oxidative stress, which results in changes in brain structure and function. There is evidence that oxidative stress regulates the long-term potentiation (LTP) signaling pathway [21]. In addition, we found an association between changes in TAS levels and improvement in cognitive factors after risperidone monotherapy. Our study suggests that some clinical manifestations, such as excitement and cognitive factors, can be predicted by plasma TAS levels and can guide psychiatrists in providing distinct treatments.

Several limitations should be noted. First, there were only two time points for data collection (baseline and week 12). It would be helpful to observe other time points on a weekly, biweekly, or monthly basis, so that more detailed trends in TAS levels over time can be evaluated. Second, we excluded substance use disorders only by asking questions regarding self-reported substance use, rather than by testing through conventional drug screening procedures. Third, only one type of antipsychotic medication was studied. The effect of different antipsychotic medications on TAS levels was not studied. Fourth, TAS levels were measured in plasma, not in the central nervous system (CNS). There are no data suggesting that changes in plasma TAS levels parallel changes in the CNS. Fifth, we did not measure the levels of inflammatory biomarkers. Considering the close relationship between oxidative stress biomarkers and inflammatory biomarkers, there is a need to further investigate the influence of TAS and inflammatory cytokines or mediators on clinical symptom reduction. Sixth, it would have been interesting to examine the relationship between TAS levels and the dose of risperidone administered in this study; however, unfortunately, we did not collect information on the dose of risperidone, which should be remedied in future studies.

## 5. Conclusions

In conclusion, higher TAS levels in patients with ANFE SCZ compared to HCs suggest that the antioxidant defense system is activated in the early stage of the pathophysiological process of SCZ patients. Risperidone monotherapy for 3 months did not normalize but further increased TAS levels in patients. Alterations in TAS levels were associated with symptom improvement in ANFE SCZ patients. Furthermore, baseline TAS levels were a predictor of clinical symptom improvement after risperidone treatment. Our findings, if replicated in future studies, will have many important clinical implications for the treatment of SCZ. They provide further evidence for predicting the efficacy of antipsychotic treatment by measuring baseline TAS levels.

## Figures and Tables

**Table 1:** Demographic characteristics and clinical data in antipsychotics-naïve first-episode (ANFE) patients with schizophrenia (SCZ) and healthy controls (mean ± SD).

Variable	Controls (*n* = 115)	Patients with TAS (*n* = 246)	**F (*p* Value)**
Sex (male/female)	64/54	138/108	0.5 (0.49)
Age (years)	27.7 ± 8.0	27.5 ± 9.2	0.03 (0.87)
Education (years)	10.4 ± 3.1	9.0 ± 3.9	12.0 (0.001)
BMI (kg/m^2^)	23.9 ± 4.5	21.3 ± 3.4	34.3 (<0.001)
Smokers/nonsmokers	42/73	67/170	2.5 (0.12)
Age of onset (years)		26.0 ± 9.1	
Baseline PANSS score			
Positive symptoms		21.8 ± 6.3	
Negative symptoms		18.8 ± 6.8	
General psychopathology		35.5 ± 9.6	
Total score		75.9 ± 17.2	

Note: BMI: body mass index; PANSS: Positive and Negative Syndrome Scale.

**Table 2:** MANCOVA analysis of TAS levels between ANFE patients and controls.

Source	Type III Sum of Squares	df	Mean Square	F	*p*
Corrected model	51,573.8	3	17,191.3	4.3	0.006
Intercept	1,020,459.1	1	1,020,459.1	252.4	<0.001
Age	21,984.4	1	21,984.4	5.4	0.02
Sex	2236.4	1	2236.4	0.6	0.46
Group	21,466.8	1	21,466.8	5.3	0.02
Error	1,350,255.0	334	4042.7		
Total	17,474,638.9	338			
Corrected total	1,401,828.8	337			

**Table 3:** Comparisons of clinical symptoms before and 3 months after risperidone monotherapy.

	Baseline	Follow-Up	t (*p* Value)
PANSS 5-factor score, mean ± SD			
Positive factor	14.7 ± 4.5	7.7 ± 3.4	22.0 (<0.001)
Negative factor	14.8 ± 6.1	12.5 ± 5.3	6.0 (<0.001)
Cognitive factor	10. ± 3.0	4.9 ± 2.2	25.0 (<0.001)
Excitement factor	8.2 ± 4.6	4.9 ± 2.1	10.1 (<0.001)
Depressive factor	5.5 ± 2.6	4.3 ± 1.8	6.8 (<0.001)
PANSS score, mean ± SD			
P subscore	22.1 ± 6.6	11.8 ± 4.5	23.3 (<0.001)
N subscore	18.8 ± 7.0	14.2 ± 5.6	10.6 (<0.001)
G subscore	35.8 ± 10.1	24.9 ± 6.0	16.6 (<0.001)
Total score	76.5 ± 18.1	50.8 ± 12.9	20.0 (<0.001)
Biomarker levels, mean ± SD			
TAS (U/mL)	216.1 ± 66.3	266.3 ± 104.8	−7.7 (<0.001)

## Data Availability

Data are contained within the article.
